# Supplementary material for: Mono-Parasitic and Poly-Parasitic Intestinal Infections among Children Aged 36–45 Months in East Nusa Tenggara, Indonesia
Source: Trop Med Infect Dis. 2023 Jan 6;8(1):45. doi: 10.3390/tropicalmed8010045 (PMC9866443; doi:10.3390/tropicalmed8010045)
Supplement: Supplementary file 1 [file tropicalmed-08-00045-s001.zip › tropicalmed-2097907-supplementary.pdf]

Supplementary Materials

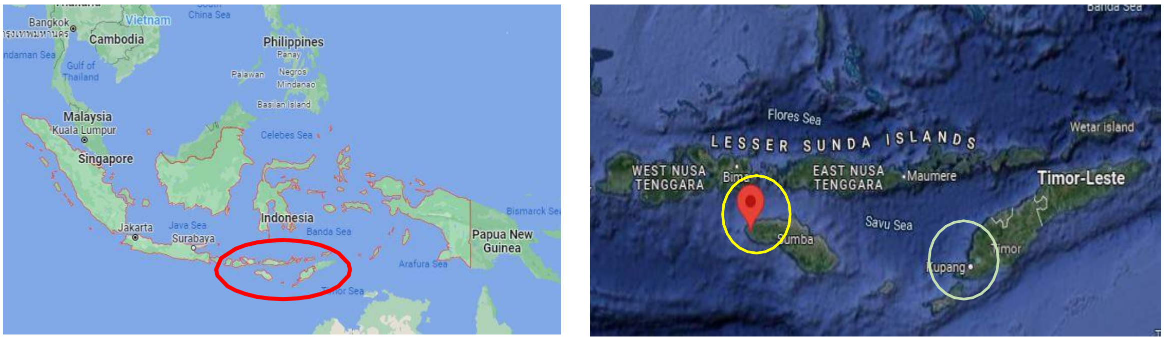

Figure S1. Location of North Kodi (yellow circle) and Kupang (green circle) in East Nusa Tenggara (red circle), Indonesia, taken from Google Earth©

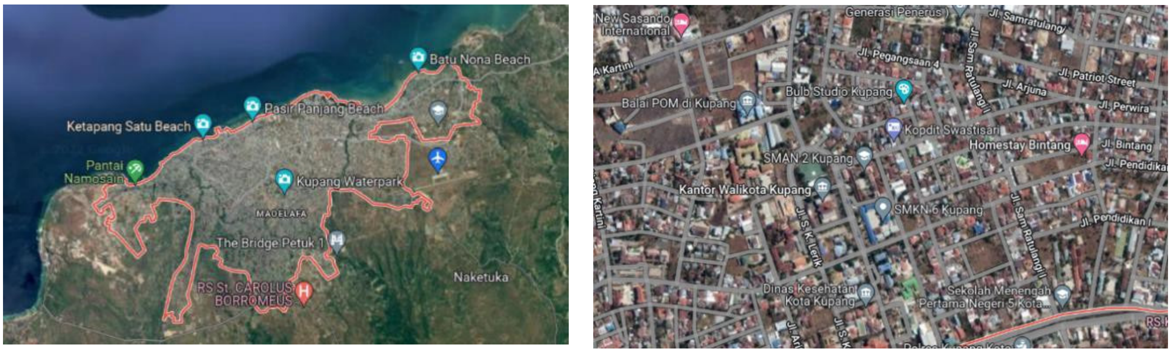

Figure S2. Kupang, East Nusa Tenggara, Indonesia, taken from Google Earth©

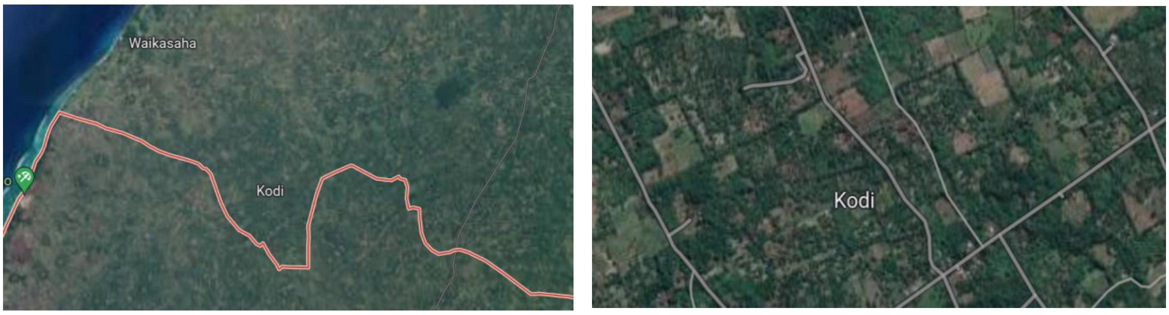

Figure S3. North Kodi District, East Nusa Tenggara, Indonesia, taken from Google Earth©

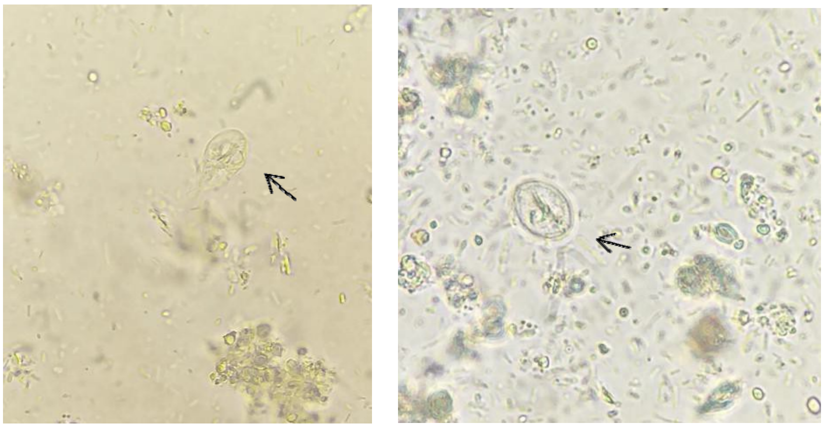

Figure S4. Direct smear of *Giardia lamblia*. *Giardia lamblia* cyst (left), *Giardia lamblia* trophozoite (right)

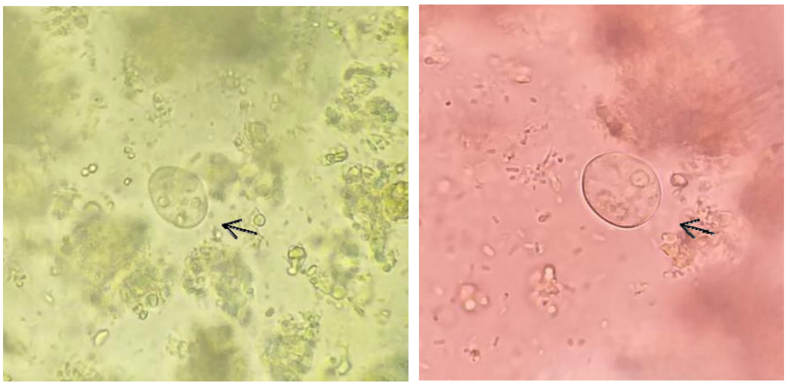

Figure S5. Direct smear of *Entamoeba Coli*. *Entamoeba coli* cyst (left), *Entamoeba coli* trophozoite (right)

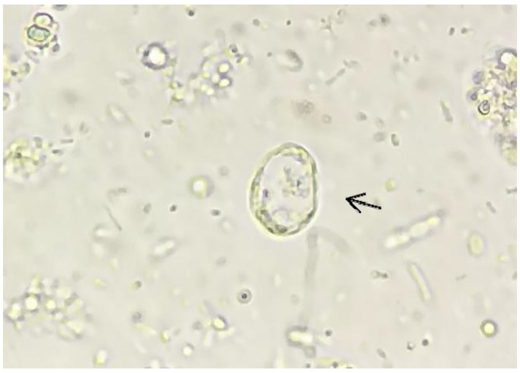

Figure S6. Direct smear of a *Blastocystis sp.* vacuole
